# A Novel Approach for Design and Manufacturing of Curvature-Featuring Scaffolds for Osteochondral Repair

**DOI:** 10.3390/polym15092129

**Published:** 2023-04-29

**Authors:** Pedro Marcelino, João Carlos Silva, Carla S. Moura, João Meneses, Rachel Cordeiro, Nuno Alves, Paula Pascoal-Faria, Frederico Castelo Ferreira

**Affiliations:** 1 Department of Bioengineering and iBB-Institute for Bioengineering and Biosciences, Instituto Superior Técnico, Universidade de Lisboa, Av. Rovisco Pais, 1049-001 Lisboa, Portugal; pmama@tecnico.ulisboa.pt; 2 Associate Laboratory i4HB—Institute for Health and Bioeconomy, Instituto Superior Técnico, Universidade de Lisboa, Av. Rovisco Pais, 1049-001 Lisboa, Portugal; 3 CDRSP-Centre for Rapid and Sustainable Product Development, Polytechnic of Leiria, Rua de Portugal-Zona Industrial, 2430-028 Marinha Grande, Portugal; carla.moura@ipc.pt (C.S.M.); joao.p.meneses@ipleiria.pt (J.M.); rachel.s.cordeiro@ipleiria.pt (R.C.); nuno.alves@ipleiria.pt (N.A.); 4 Associate Laboratory for Advanced Production and Intelligent Systems (ARISE), 4050-313 Porto, Portugal; 5 Polytechnic Institute of Coimbra, Applied Research Institute, Rua da Misericórdia, Lagar dos Cortiços—S. Martinho do Bispo, 3045-093 Coimbra, Portugal; 6 Veterinary Clinics Department, Abel Salazar Biomedical Sciences Institute, University of Porto, Rua de Jorge Viterbo Ferreira 228, 4050-313 Porto, Portugal; 7 Department of Mechanical Engineering, School of Technology and Management, Polytechnic of Leiria, Morro do Lena—Alto do Vieiro, Apartado 4163, 2411-901 Leiria, Portugal; 8 Department of Mathematics, School of Technology and Management, Polytechnic of Leiria, Morro do Lena—Alto do Vieiro, Apartado 4163, 2411-901 Leiria, Portugal

**Keywords:** 3D printing, curvature-featuring scaffolds, finite element modelling, mechanical properties, osteochondral regeneration, tissue engineering

## Abstract

Osteochondral (OC) defects affect both articular cartilage and the underlying subchondral bone. Due to limitations in the cartilage tissue’s self-healing capabilities, OC defects exhibit a degenerative progression to which current therapies have not yet found a suitable long-term solution. Tissue engineering (TE) strategies aim to fabricate tissue substitutes that recreate natural tissue features to offer better alternatives to the existing inefficient treatments. Scaffold design is a key element in providing appropriate structures for tissue growth and maturation. This study presents a novel method for designing scaffolds with a mathematically defined curvature, based on the geometry of a sphere, to obtain TE constructs mimicking native OC tissue shape. The lower the designed radius, the more curved the scaffold obtained. The printability of the scaffolds using fused filament fabrication (FFF) was evaluated. For the case-study scaffold size (20.1 mm × 20.1 mm projected dimensions), a limit sphere radius of 17.064 mm was determined to ensure printability feasibility, as confirmed by scanning electron microscopy (SEM) and micro-computed tomography (μ-CT) analysis. The FFF method proved suitable to reproduce the curved designs, showing good shape fidelity and replicating the expected variation in porosity. Additionally, the mechanical behavior was evaluated experimentally and by numerical modelling. Experimentally, curved scaffolds showed strength comparable to conventional orthogonal scaffolds, and finite element analysis was used to identify the scaffold regions more susceptible to higher loads.

## 1. Introduction

The human joints are dynamic complex structures responsible for providing a near-frictionless interface between bones and allowing for constrained coordinated motion [1,2]. In diarthrodial joints (e.g., knee joint), articular cartilage and subchondral bone form a composite structure designated as the osteochondral (OC) unit, which comprises a complex and fine interplay between its components under physiological conditions [3].

Two types of OC defects can be described according to the phenotype. Focal lesions are generally well delineated and usually caused by trauma or illnesses, such as osteochondritis dissecans or osteonecrosis. Degenerative lesions are attributed to progressive deteriorating changes to the structures of the joints, resulting, for example, from ligament instability, meniscal tears, or OC unit diseases such as osteoarthritis (OA) [4]. These diseases create a very high socioeconomic burden on national healthcare systems. OA, the most common joint disease, is estimated to affect over 250 million people worldwide, and its prevalence is expected to continue to increase [5]. Among the consequences of OA are the loss in mobility and performance limitations in daily activities, with an associated cost, including direct medical expenses and indirect expenditures due to the loss in productivity, estimated to be between 1% and 2.5% of the Gross Domestic Product in high-income countries [6].

Regardless of the origin, damage in the OC unit leads to a cascade of events attempting to repair the injury. However, due to its avascular nature, these self-healing processes are very limited in articular cartilage, often leading to the formation of scar tissue of inferior quality. In the long run, the inadequacy of this scar tissue will likely lead to an increase in the severity of the defect, which will reach the subchondral bone [7]. Therefore, the repair of both components of the OC unit should be therapeutically addressed, since they are physiologically deeply interconnected. This presents a major challenge due to the intrinsically different properties and healing capacities of the articular cartilage and subchondral bone [4]. Therapeutic options for OC repair are selected based on the size and severity of the defect [8]. Currently available treatments range from non-operative conservative strategies, managing the symptoms and reducing risk factors for the less severe cases, to increasingly extensive surgical interventions such as arthroscopic lavage and debridement, abrasion arthroplasty, microfracture, OC autografting and allografting, whole-joint replacement surgeries, and also cell-based procedures such as autologous chondrocyte implantation (ACI) and matrix-induced autologous chondrocyte implantation (MACI), possibly combined with growth factors [9,10]. However, all of these methods still fail to fully restore tissue structure and properties, compromising long-term clinical outcomes [11,12].

Tissue engineering (TE) has been proposed as a promising therapeutic alternative for producing OC tissue substitutes. These strategies involve the proper combination of cells, biomaterial scaffolds, and external stimuli in the form of biochemical (e.g., growth factors and cytokines) and physical factors (e.g., mechanical or electrical stimulation) provided by bioreactor culture systems [13,14,15,16]. Among these components, scaffolds play an important role in supporting cell proliferation and differentiation and extracellular matrix production. In an ideal case, they should emulate structurally and functionally the native tissue, providing a biocompatible and biodegradable environment with a degradation rate compatible with the formation of newly regenerated tissue [17,18]. Requirements can also consider other important aspects related to the following: the scaffold structure, providing a hierarchical organization with proper porosity and interconnectivity that enables cell migration and nutrient and waste diffusion, tailored to the target tissue and cells; the scaffold functionality, giving the ability to interact with host cells and integrate with host tissue by incorporation of growth factors and biological cues and exhibiting native-like biomechanical properties; and the fabrication method, which should be precise, easily scalable and reproducible, and versatile to accommodate individualized “patient-tailored” variations in the construction [11,19,20,21,22].

The available scaffold fabrication technologies can be grouped into two main categories: conventional and additive manufacturing (AM) methods. Conventional techniques use mostly subtractive methods, in which parts of a material are removed to obtain the final conformation, while in AM technology, the final construction is obtained by successive deposition of overlaying layers [23]. Methodologies such as solvent casting/particle leaching, freeze-drying, gas foaming, phase separation, and electrospinning are included in the conventional techniques, while stereolithography, selective laser sintering, inkjet 3D printing, and extrusion bioprinting are defined as AM technologies [24]. Some advantages are recognized to the AM techniques when compared to the conventional methods, such as scalability and reproducibility; high versatility, as they can use a wide range of materials; higher control over scaffold geometry, predictable and consistent at the macro- and micro-scale; the possibility of fabricating more complex structures, more easily adaptable to create patient-specific scaffold designs; and avoiding the use of organic solvents, which are required in some conventional techniques and could compromise cell viability and the biological performance of the scaffolds [23,25,26,27,28]. For these reasons, AM methods have been increasingly used for manufacturing scaffolds for different TE applications [25].

A common AM-based method for scaffold fabrication is material extrusion, owing to its accessible cost and ability to work with a wide range of materials, which has found applications in the fabrication of blood vessel, bone, cartilage, neural, cardiac, skeletal muscle, liver, and skin TE constructs [29,30,31,32,33]. These systems incorporate fused filament fabrication (FFF) using thermoplastic polymers and composites, to which cells and biochemical factors may be added after scaffolds construction [34,35,36,37]. The used materials can be grouped into synthetic polymers (e.g., poly(ethylene glycol) (PEG), poly(lactic acid) (PLA), Poly(lactic-co-glycolic acid) (PLGA), Poly(ϵ-caprolactone) (PCL), Poly(propylene fumarate) (PPF)), bioceramics (e.g., hydroxyapatite (HAp), tricalcium phosphate (TCP)), natural polymers (e.g., hyaluronic acid, chondroitin sulphate, alginate, agarose, collagen, gelatin), and extracellular matrix (ECM)-based materials (e.g., decellularized ECM, pulverized ECM particles) [11,19,38,39,40]. A key factor, typically considered in choosing the scaffold materials for OC TE applications, is the purpose of having constructs that can approach the mechanical properties of articular cartilage and bone to achieve the natural load-bearing properties of the native OC tissue [41].

In the context of OC TE, scaffold design has seen a significant evolution in architectural complexity, aiming to achieve a greater resemblance to the structure of the native tissue. The field evolved from scaffolds consisting of monophasic constructs to biphasic constructs, recognizing the distinction between articular cartilage and the subchondral bone; to triphasic constructs, realizing the significance of the calcified cartilage between articular cartilage and the subchondral bone; and finally to multiphasic and gradient constructs, in an attempt to mimic the native tissue hierarchical gradient structure [11,42]. However, despite this evolution, most described scaffolds still exhibit a cuboid or cylindrical shape, which could lead to a mismatch in geometry at the interface between the tissue and the scaffold, resulting in stress concentrations. This may hamper the engineered host tissues’ integration, accelerate weakening of the scaffold structure, or increase local stress in the surrounding tissue [42,43]. Furthermore, the filament placement strategy used in extrusion fabrication systems has not seen a significant change from the successive deposition of overlaying of layers, which has mostly been dictated by the commonly available AM fabrication algorithms [44,45]. Nevertheless, some exceptions have been reported using extrusion, reproducing anatomical shapes, such as the articular surface of a rabbit synovial joint [46], the shape of an ear [47], a calvarial reconstruction [48], and a human vertebral body [49], but these cases are still greatly outnumbered.

Several key aspects have been identified as of significant relevance for the successful culture of living cells within scaffolds and bioreactor devices for TE strategies. For example, the interactions of cells with the surrounding ECM and other cells, as well as the influence of environmental factors such as mechanical (e.g., flow-induced effects, compressive loading), electrical, and biochemical stimuli, have been shown to modulate cell proliferation, differentiation, and metabolic functions [50,51]. As a complement to the in vitro experimental research on the influence of these aspects, the use of detailed mathematical models as a virtual in silico representation of the tissues/scaffolds/bioreactors can be advantageous. These models are designated digital twins and are developed to provide a more profound characterization of the culture systems/scaffold features, optimizing stimulation parameters and predicting experimental outcomes while reducing the time and costs involved [4,15,52,53]. 

In this work, we present a novel methodology for the design and rapid manufacturing of scaffolds to mimic the native curvature of OC tissue. The scaffold curvature was defined mathematically, and an automated parametric design process was implemented for the construction of scaffolds with the desired curvatures using computer-aided design (CAD) software. The FFF technique was chosen to produce the scaffolds, and a procedure was devised to determine the highest curvature this method could produce. Moreover, a structural analysis by micro-computed tomography (μ-CT) and scanning electron microscopy (SEM) was conducted to assess the shape fidelity of the printed scaffolds. Finally, due to its relevance in the context of OC repair strategies, the mechanical properties of the produced curvature-featuring FFF-based scaffolds were evaluated by uniaxial compressive testing and studied in more detail using finite element analysis (FEA).

## 2. Materials and Methods

### 2.1. CAD Modelling, Characterization, and Fabrication of Scaffolds

Scaffold models were created with the CAD software Autodesk Fusion 360, up to version 2.0.11415, automatically updated online (Autodesk, Inc., San Francisco, CA, USA). One advantage of CAD packages is parametric design. Concerning scaffold modelling, this allowed us to create scaffolds using the built-in geometric operations, and by assigning distinct values to only one parameter, which controls the curvature, we were able to use the same workflow to automatically create as many distinct curved scaffolds as needed. The detailed description of this modelling workflow is provided in Section 3.1.1. Common to all scaffold models, a fibril (the term used in this manuscript to designate the tube-like strands in the CAD drawings) with a diameter of 300 μm and a layer height of 300 μm was defined.

For the CAD models, the porosity was obtained through the following Equation (1):

(1) $$Porosity=\left(1-\frac{V_{solid}}{V_{total}}\right)\times 100\%$$

where *V_solid_* and *V_total_* are, respectively, the volume occupied by the solid fraction and the total volume in a volume of interest.

To create objects suitable for 3D printing, the models were exported from Fusion 360 as STL files with high refinement. The 3D printing was executed by FFF with a Prusa i3 MK3S commercial 3D printer (Prusa Research, Praha, Czech Republic), to which a 0.25 mm brass nozzle (Prusa Research) was adapted, instead of the standard 0.4 mm nozzle. The prints were made from a 1.75 mm spooled PLA filament from Velleman (Velleman Group nv, Gavere, Belgium). Furthermore, 3D-printer-readable G-code files were created using PrusaSlicer 2.3.1 (Prusa Research). A printing layer height of 0.15 mm was defined for all layers (since the 300 μm scaffold fibril height would be a dimension too big to print in a single run with a 0.25 mm nozzle) and an extrusion width of 0.3 mm. The printing temperature was set to 210 °C and the bed temperature to 65 °C. Printing speed was adjusted according to the features of the construct being produced. For external and internal perimeters of parts, speeds of 20 mm/s and 30 mm/s were employed, respectively. For infill and travel movements, a speed of 45 mm/s was employed. Depending on the applicability to the features of the construct being printed, the additional parameters were set: 100% infill density; rectilinear infill pattern with alternating -45° and 45° raster orientations; and 3 perimeters.

### 2.2. Micro-Computed Tomography (μ-CT) Analysis

The microstructure of the scaffolds was evaluated by μ-CT with a SkyScan 1174v2 instrument, Bruker version 1.1 (Bruker, Billerica, MA, USA). Image reconstruction was performed using NRecon version 1.7.4.6 (Bruker), and CTVox version 3.3.1 (Bruker) and CTVol version 2.3.2.0 (Bruker) were employed to obtain realistic 3D visualizations of the scanned scaffold samples. CTAn version 1.20.0 (Bruker) was used for the reconstruction analysis. The following acquisition parameters were used: source voltage of 50 kV; source current of 800 mA; image pixel size of 30.11 μm; exposure time of 9000 ms; rotation step of 0.5°; frame averaging on (3); no filter.

### 2.3. Scanning Electron Microscopy (SEM) Imaging

The surface morphology of the printed curved scaffolds was evaluated by SEM analysis with a Hitachi S2400 SEM instrument (Hitachi, Ltd., Tokyo, Japan) operating at 20 kV acceleration voltage. Prior to scanning, scaffold samples were sputter-coated with a thin layer of gold/palladium by a Q150T ES sputter coater (Quorum Technologies, Laughton, East Sussex, UK).

### 2.4. Compressive Mechanical Testing

The experimental structural behavior of the parts was assessed, under compressive mechanical loading, using an Instron 4505 machine (Instron, Norwood, MA, USA) equipped with a 100 kN load cell and applying a constant displacement rate of 1 mm/min. Seven specimens were used for each scaffold condition analyzed (*n* = 7). The compressive modulus was determined from the slope of the initial linear regions of the stress–strain curves. Yield strength was calculated using the offset yield method, with the offset line parallel to the modulus line and a displaced strain of 0.2%. The yield strength was identified as the point of intersection between the offset line and the stress–strain curves.

Due to the curvature of the scaffolds created, an assembly of blocks joined with the scaffolds was designed and 3D-printed to allow the performance of the compressive tests. A description of the design approach is presented in Section 3.1.3. Supplementary Figure S1 shows the designs of the parts manufactured and tested mechanically under compressive loading.

### 2.5. Finite Element Analysis

Finite element analysis (FEA) was performed using the Solid Mechanics module from COMSOL Multiphysics 5.2a software (COMSOL Inc., Stockholm, Sweden). A stationary study was conducted to determine the stresses when scaffolds are subjected to compressive loadings. 

Models were created in Fusion 360 and imported into COMSOL as STEP files. To emulate the experimental compression testing, the imported models had a geometry equivalent to the geometry of models described in Section 2.4. Taking advantage of the existence of two planes of symmetry, the imported models result from sectioning along these two planes to facilitate the numerical computer calculations (Supplementary Figure S2). These planes of symmetry can be defined in COMSOL as boundary conditions, and the simulation results correspond to the compression of the whole models. The scaffold and block assemblies were defined as single domains in COMSOL. The flat surface on one side was considered fixed, while a displacement of 0.4 mm was prescribed to the flat surface on the opposing side (Supplementary Figure S2). This displacement corresponds approximately to a strain obtained from the experimental compression results described in Section 2.4 when a transition is identified from elastic to plastic behavior. As a result, in the implemented COMSOL simulations, the material model contemplated just the isotropic linear elastic behavior. For all simulations, the assemblies were specified to be constituted by PLA. A range of values is reported in the literature for the density and Poisson’s ratio for PLA samples. Accordingly, this study was performed considering a density of 1.24 g/cm^3^ [54] and a Poisson’s ratio of 0.3 [55]. Concerning the Young’s modulus, the value determined experimentally in the compression of the 100% infill block, made of the same PLA used in scaffold fabrication, was chosen as the most appropriate for this material characteristic, as discussed in Section 4. The mesh elements for all models were created with a tetrahedral geometry and using the physics-controlled element size definition, in which the option “finer” for the element size parameter was chosen (element sizes in the range 0.1 mm to 1.38 mm). Considering all models, the skewness average element quality was always above 0.6627. The numerical model predictions were interpreted using the calculated von Mises stresses. Plots were obtained for the entire volume of the domains and along lines crossing the entire length of the scaffolds.

### 2.6. Statistical Analysis

The results are presented as mean values ± standard deviations (SD) when applicable. The statistical analysis was performed using GraphPad Prism 7.0 (GraphPad Software, San Diego, CA, USA). To assess statistically significant differences between independent samples, ANOVA tests were performed, followed by Tukey’s multiple comparison test (for significant *p*-values, * denotes *p* < 0.05, ** denotes *p* < 0.01, *** denotes *p* < 0.001, **** denotes *p* < 0.0001).

## 3. Results

### 3.1. Scaffold Design Methodology and Assumptions

#### 3.1.1. Curved Scaffold Design Procedure

**Figure 1:**
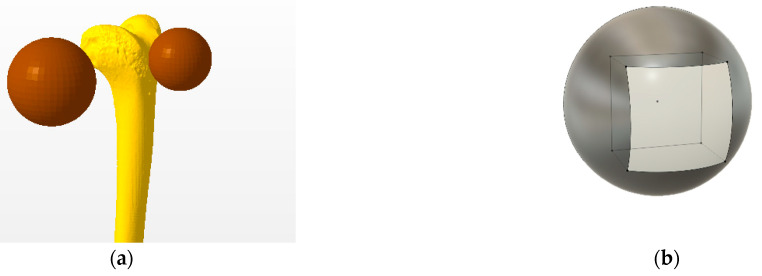
(**a**) Representation of matching the curvature of the femoral condyles to specific sphere sizes, as a strategy for designing curved scaffolds. (**b**) Illustration of fitting a square segment on the surface of a sphere, with the resulting section to be used as a template for the design of the curved scaffolds.

The motivation for developing curved scaffolds was the nonplanar native structure of tissues in the human musculoskeletal system, particularly of the OC tissue in the knee joint, which can be affected by highly debilitating conditions such as OA. Figure 1a shows the distal femur and two spheres that approximate the native curvature of both its condyles. As a first approach, a sphere was chosen for simplicity, as it only requires a radius to be defined and modelled. Furthermore, from a manufacturing perspective, with the accessible FFF printing technology, it was convenient that the scaffold would have straight sides, therefore averting the use of supporting structures connected to the build plate during the printing process. Accordingly, Figure 1b represents the square-shaped section made on the surface of spheres that formed the template for the design of the scaffolds.

**Figure 2:**
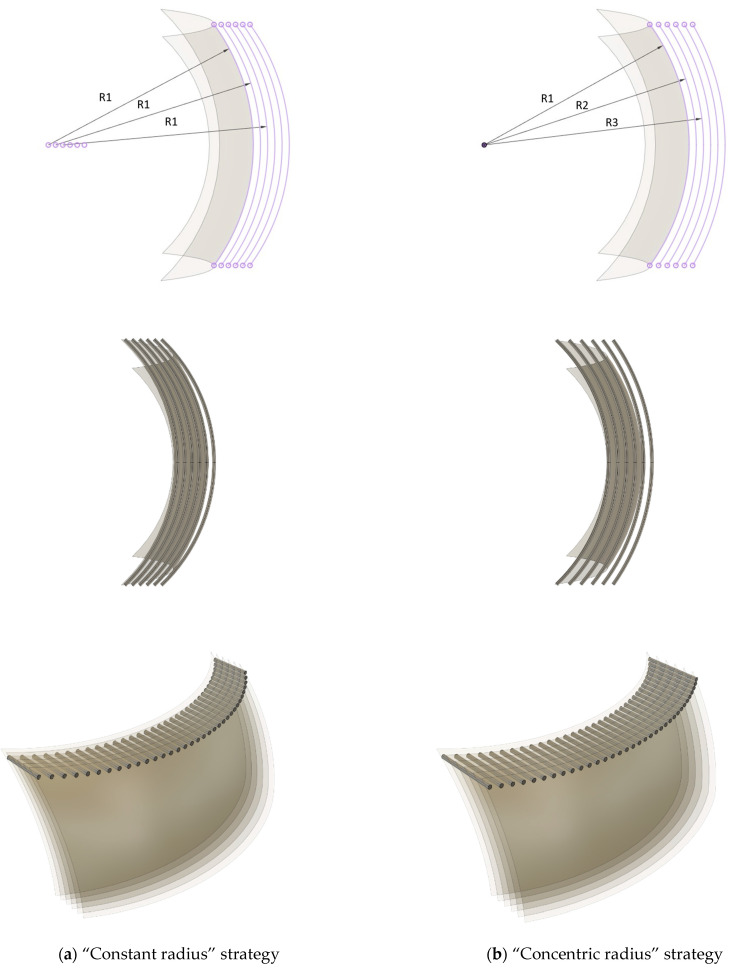
Strategies for curved scaffold design: (top) Shaded square-shaped section of a spherical surface and arcs resulting from the intersection with a plane. (**a**) In the “constant radius” strategy, equal arcs are obtained from a plane intersecting translated sections (all radii have the same value R1); (**b**) in the “concentric radius” strategy, successively larger arcs are obtained from the plane intersecting concentric sections (R1 < R2 < R3). (center) Illustration, on the top layer, of the curved fibrils drawn following the arcs. (bottom) Illustration of the curved fibrils; the straight fibrils are designed to connect the two farthest-apart spherical sections.

Using conventional slicing algorithms, each layer of the scaffold would be printed at a time, and each successive layer would be printed on top of the previously deposited material. This strategy also carried the advantage of having fewer interruptions in filament flow during printing, resulting in parts with improved structural stability and mechanical strength. To have scaffolds with a grid-like pattern and, therefore, a porous structure, the design would have fibrils patterned with two alternating orientations: (i) one with curved filaments, following the intersection of the square-shaped sections with planes at established layer heights; (ii) the other with straight filaments, alternating with the curved ones (Figure 2). Two strategies were applied to define the location of the curved fibrils. In the designated “constant radius” strategy, the square-shaped section was translated to a distance corresponding to the sum of fibril and pore widths the number of times necessary to have the desired thickness in the scaffold (Figure 2a). In the “concentric radius” strategy, a square-shaped section was made in a circle with a specified radius. Then, circles with the same center position but with increasingly larger radii were drawn, and sections of those circles defined the locations of the curved fibrils (Figure 2b). A distinctive consequence of modelling according to these two strategies can be visible towards the edges of the scaffolds. With the “constant radius” strategy, the porosity will decrease, while with the “concentric radius” strategy, the porosity will increase. Without an anticipated advantage of one over the other, both strategies were used in the subsequent stages of the curved scaffolds design.

**Figure 3:**
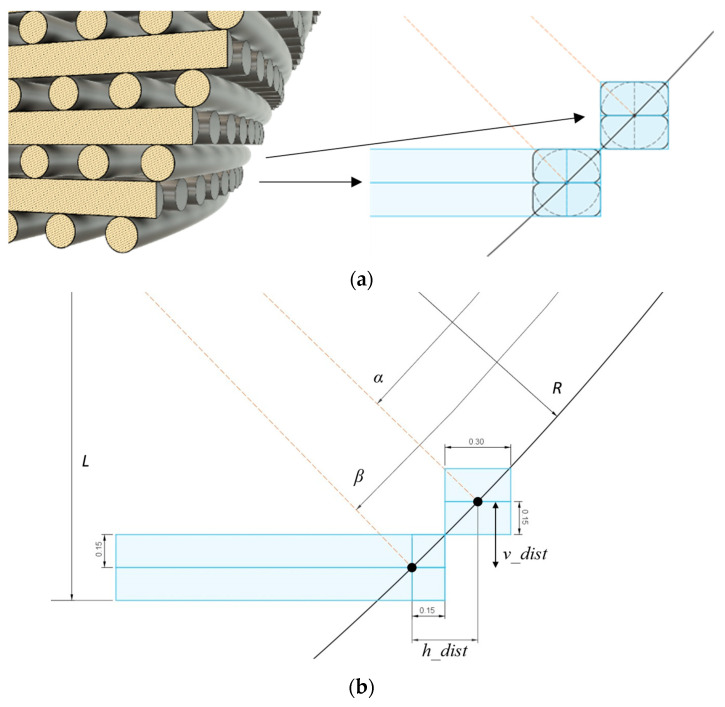
Printability of scaffolds: (**a**) Representation of the limit criteria assumed for the printability of a scaffold by FFF. There should be, at least, a tangential contact between successive layers. (**b**) CAD representation of a cut through the bottom layer of a scaffold, where the limit tangential contact will manifest. A system of equations was set up to relate the indicated parameters: the horizontal (*h_dist*) and vertical (*v_dist*) distances between the two black dots (the intersection of successive filament layers with the sphere section defining the curvature), the radius of the sphere section (*R*), the distance from the center of that sphere to the bottom of the scaffold (*L*), and the angles *α* and *β* measured from the center of the sphere.

For the effectiveness of printing with FFF, the material being deposited should be supported by the material already in place to have the parts match the desired geometry. More visible deformations can be seen when the deposited filament has to bridge across larger spans or the deposited unsupported sections are farther from being connected in a straight line. The decision to not use support structures or material was also made because this leads to parts with inferior surface quality and properties due to scarring resulting from the supports’ removal. Therefore, a limit was imposed for each layer to be supported solely by already-deposited material. Due to the scaffold curvature and the path already determined for the filament deposition, the least supported locations would be noticeable in the first and last printing layers, where there is a steeper slope between the layers (Figure 3a). Therefore, the design objective became to calculate the model parameters, which led to the limit of tangential support between successive layers. This limit could not be exceeded for a print to be considered successful.

The relevant parameters established to calculate the curvature limit are indicated in Figure 3b, which shows a cut through the bottom half of a sphere’s square-shaped section and the fibrils deposited in the first four printed layers. The black dots denote the intersection of the section with the layer heights defining the position of the deposited filaments. All parameters are related in a system of equations (Equation (2)) that describes the following: (i) the difference in horizontal distance between the two dots (*h_dist*), (ii) the difference in vertical height between the two dots (*v_dist*), and (iii) the height of the designed scaffold, relative to its midplane (*L*). Here, *R* represents the radius of the sectioned sphere, and *α* and *β* the angles from the midplane to the filaments’ positions, considering as vertex the sphere’s center.

(2) $$\left\{\begin{array}{c} h\_dist=R\left(\cos\alpha -\cos\beta \right)\\ v\_dist=R\left(\sin\beta -\sin\alpha \right)\\ L-0.15=R\sin\beta \end{array}\right.$$

Figure 3b and Equation (2) indicate some dimensions pertinent to the scaffold design and printing process. In TE scaffolds, pore size is one of the most relevant parameters influencing cell proliferation, differentiation, tissue formation, and vascularization, and it has been used as a means to elicit the desired biological response [56]. Therefore, to have dimensions compatible with OC TE constructs, both the fibril diameter and the pores were set to have a width of 300 μm, consistent with dimensions previously described in the literature [57,58]. Regarding the pores, this dimension was measured at the center of the scaffold. This choice considers the fact that for both design strategies, the pore size is the same at the center but varies towards the edges of the scaffolds, with the porosity increasing in the “concentric radius” design and decreasing in the “constant radius” design. As stated in Section 2.1, each filament was split into two printhead passages, represented by the blue line splitting the fibrils horizontally.

The system in Equation (2) can be solved for the desired variables, if sufficient parameters have been assigned. For example, given a height *L* for the scaffold and assuming a tangential contact between the first layers, the system can be solved for the radius *R* and the angles *α* and *β*. Conversely, if a radius is assigned to match the curvature of a tissue, and a dimension *L* is assigned to the scaffold, the system can be solved for *h_dist*. Thus, a conclusion could be reached about whether this scaffold architecture would be self-supported during the printing process. Given a scaffold height, the system in Equation (2) can be solved for the radius *R*. The lower the *R* value is, the more curved the scaffold obtained. However, there is a lower limit for its value, imposed by printability. This represents the situation, previously mentioned as limit of tangential support, in which scaffold integrity is compromised due to lack of support between successive layers, typically observed towards the sides of the scaffold.

**Figure 4:**
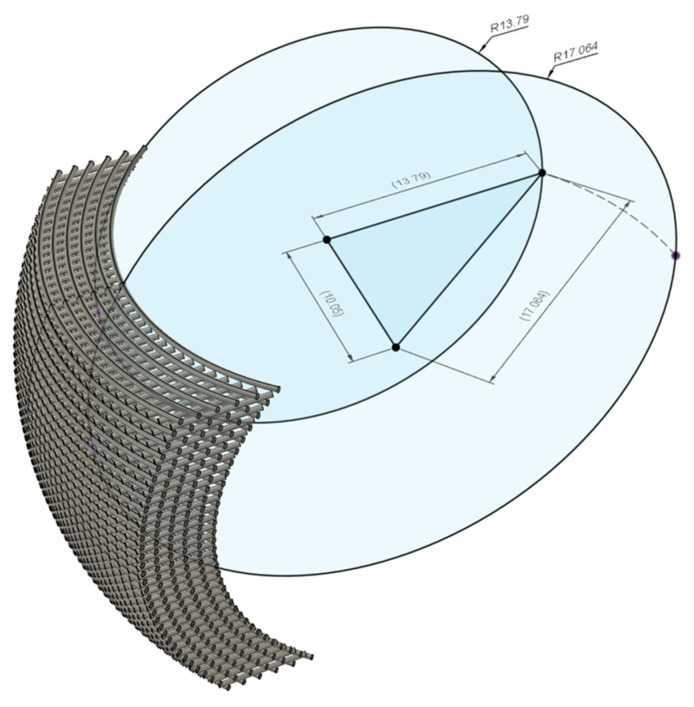
Determination of radius through the center of the scaffold. This radius is used to define the curvature in CAD, as a parameter set to define the curvature independent of the scaffold height and width.

Defining the curvature with a parameter independent of the scaffold height and width was more convenient for design and comparison purposes. For this reason, the radius through the center of the scaffold was calculated (Figure 4). Then, a scaffold can be designed with a specific curvature and later verified if it features unsupported fibrils. Thus, if the width or height of the scaffolds were to be modified, the overlapping sections would still have the same geometry. In Table 1, a summary of the scaffold dimensions and calculated parameters is presented.

**Figure 5:**
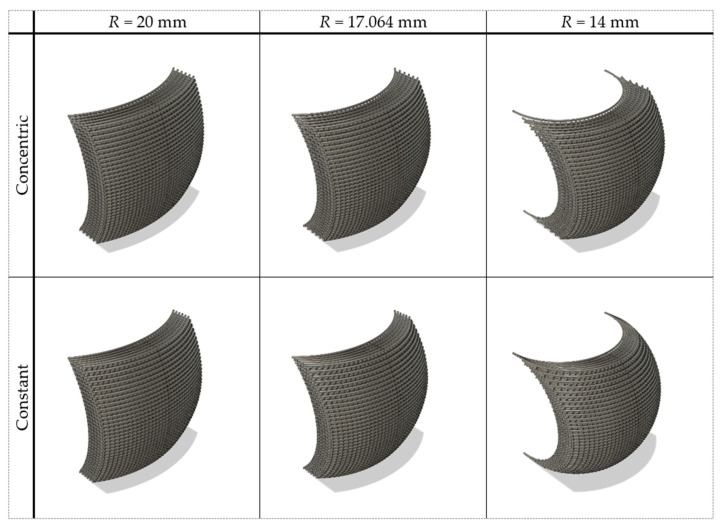
CAD models of the designed curved scaffolds. Designs for both strategies, “concentric radius” and “constant radius”. The chosen radii (*R*) correspond to the determined limit of printability and values over (20 mm) and below (14 mm) that limit.

To compare and verify the supported material printability assumptions taken in model definition, scaffolds were designed with the limit-determined curvature and with greater and smaller curvatures, using both “constant radius” and “concentric radius” design strategies. The greater and smaller curvature radii were chosen so that both had a similar difference concerning the limit printability radius and, with the smaller radius, the square section would be close to intersecting the projection of the sphere. This resulted in modelling scaffolds with 14 mm and 20 mm curvature radii. The developed CAD models are represented in Figure 5.

#### 3.1.2. Orthogonal Scaffold Design

An orthogonal scaffold was also designed with the same top projected dimensions as the curved scaffolds, i.e., the same side lengths of the square used to achieve the sectioning of the sphere. The side on which it was printed was equivalent to those used to print the curved scaffolds. As for the curved scaffolds, pore size and fibril width were defined as 300 μm. A representation of this scaffold is shown in Supplementary Figure S3.

#### 3.1.3. Block Design for Mechanical Testing under Compressive Loading

Since mechanical testing equipment compresses parts between two flat plates, the curved scaffolds could not be subjected directly to this test, given that the load would be transmitted only between the contact points of the curved scaffold with the plates instead of the whole surface of the scaffold. For this reason, solid blocks were modelled and added to all sides of the scaffolds so that they would, on one side, follow the curvature of the scaffolds and, on the other, have the proper flat surface necessary for the compression tests. The models included a slight overlap between the blocks and the scaffold, and in the 3D-printing process, they were printed as one single part to avoid slippage during testing. In all cases, the height of these assemblies was designed to be 25 mm, with the contact compression area corresponding to the square area used to achieve the sectioning of the sphere. The models created for each scaffold condition are shown in Supplementary Figure S1. Additionally, blocks integrating the orthogonal scaffold and solid blocks with the same global dimensions were also designed for comparison.

### 3.2. Scaffold Structural Characterization

**Figure 6:**
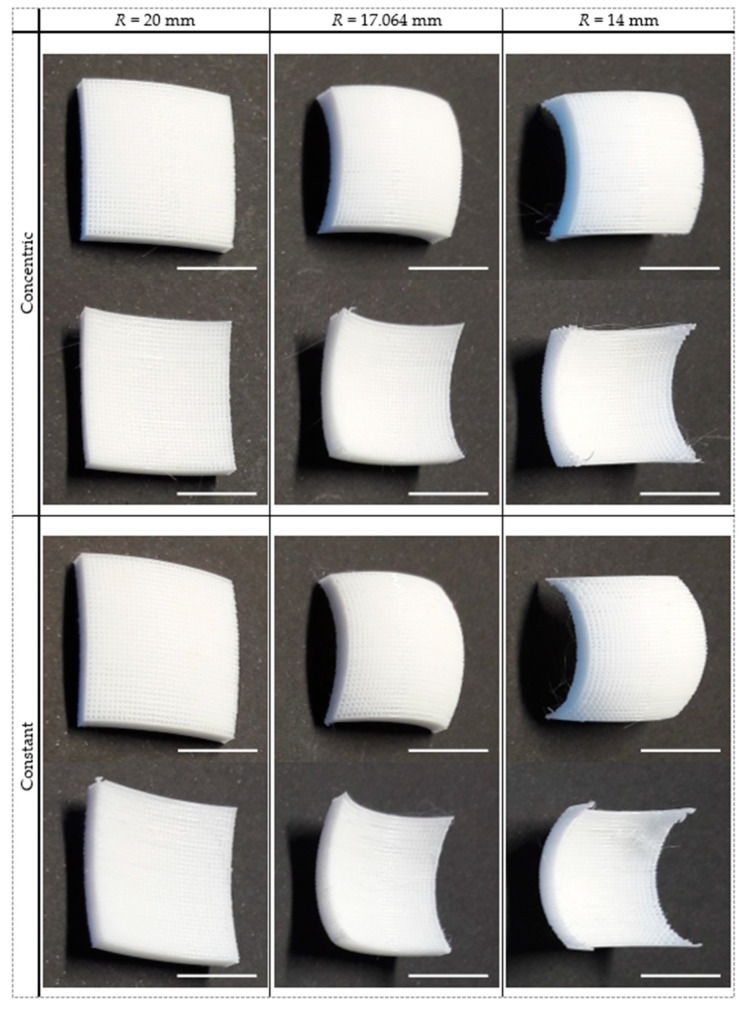
Examples of FFF-printed curved scaffolds based on the CAD models. Scaffolds were printed using both design strategies: “concentric radius” and “constant radius”. The chosen radii (*R*) correspond to the determined limit of printability (17.064 mm) and values over (20 mm) and below (14 mm) that limit (scale bars: 1 cm).

**Figure 7:**
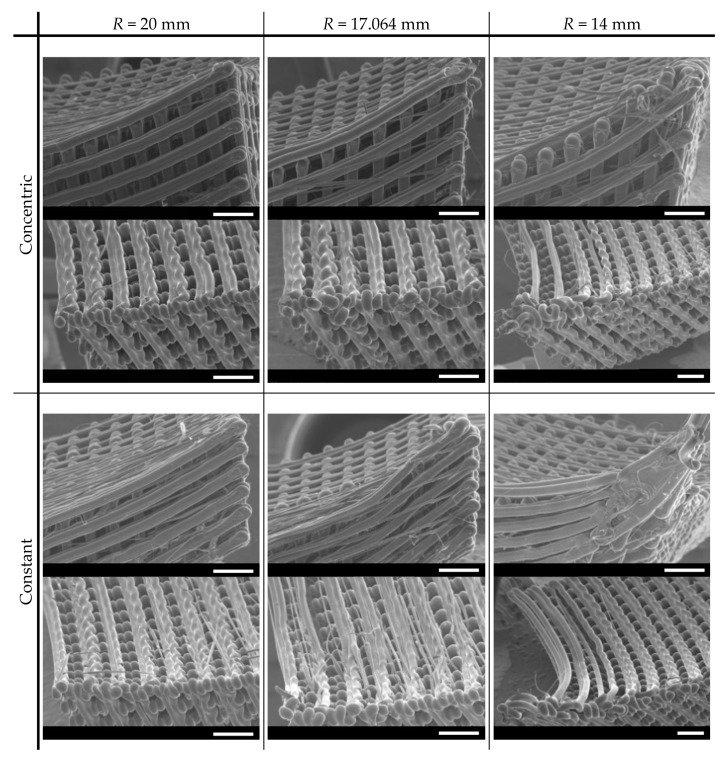
SEM imaging in the corners of the scaffolds using both design strategies, “concentric radius” and “constant radius”. The chosen radii (*R*) correspond to the determined limit of printability (17.064 mm) and values over (20 mm) and below (14 mm) that limit (scale bars: 1000 μm).

**Figure 8:**
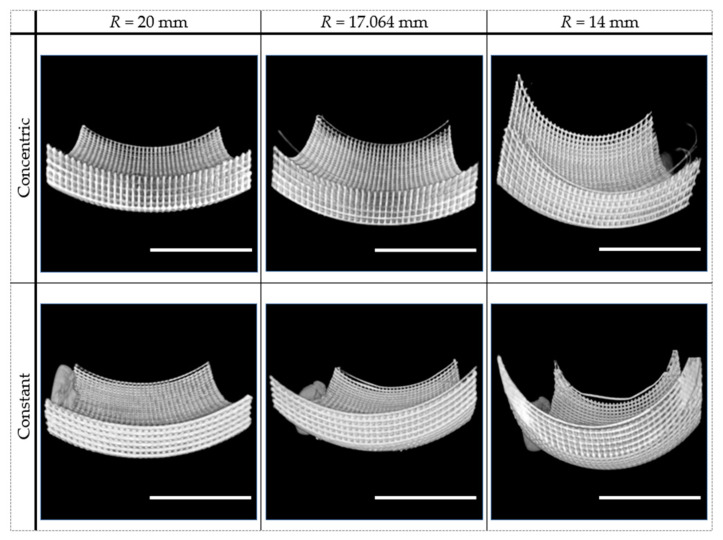
Representation of the μ-CT 3D reconstructions of the curved scaffolds using both design strategies, “concentric radius” and “constant radius”. The chosen radii (*R*) correspond to the determined limit of printability (17.064 mm) and values over (20 mm) and below (14 mm) that limit (scale bars: 1 cm).

Curved scaffolds were printed using PLA by FFF, with a curvature defined by the radius of a sphere and with projected dimensions of 20.1 mm × 20.1 mm (Figure 6). For structural and shape fidelity analyses, the scaffolds were assessed through SEM imaging (Figure 7) and by μ-CT analysis (Figure 8). Figure 7 specifically details the corners, where the more severe material detachment was predicted and observed. For the 20 mm curved scaffolds, obtained following both design strategies, the filaments were printed in the expected location, and no detachments were observed. The division of each fibril into two distinct layers is clearly visible. In the curved fibrils, a wavy pattern is seen in the bottom printed layer, but a connection to the straight fibrils is still clearly visible. For the scaffolds with a radius of 17.064 mm, the curvature of the top fibrils shows some deviation from a circular profile, suggesting a poor adhesion in relation to the fibrils printed underneath, with the filament being dragged out from the designed position. Some filaments are also clearly detached in some of the scaffold in the “constant radius” strategy. For the 14 mm scaffolds, the top curved layers are completely detached from the scaffolds for both design strategies. In particular, in the “concentric radius” strategy, one top curved fibril is even missing, since it had very limited contact points with the other parts of the scaffold. In the “constant radius” strategy, the change in position of discrete fibrils in a surface can be seen in the corners of the top layer. Due to the overlap, the slicing algorithm joined the extremities of the fibrils into the observed surface.

Overall, in the scaffold corners, an increase is observed in imprecisely deposited filament with the decrease in the radius, due to material deposition without support. Another printing artifact, also observed in Figure 7, is stringing, resulting from release of the residual pressure inside the nozzle between deposition movements. Both are artifacts that impact scaffold geometry and properties.

In Figure 8, the difference between the two design strategies is clearly perceptible from the images shown. In the “concentric radius” strategy, scaffolds are thinner at the center and become wider towards the extremities. Additionally, by decreasing the radius, this enlargement in the extremities becomes even more noticeable. Conversely, in the “constant radius” strategy, a width reduction is observed towards the sides of the scaffolds, and such reduction is more perceptible with the decrease in the radius. For both strategies, no detachments were observed for the 20 mm radius, neither on the top nor on the bottom layers, the locations where misplacement of filament deposition during printing is more challenging. However, for the 17.064 mm and 14 mm radii, detachment of some fibrils is observed in the top and bottom printed layers. For the scaffolds made following the “concentric radius” strategy with radii of 17.064 mm and 14 mm, the fibrils were printed with the correct shape. This result suggests that the minimal contact with the previously deposited layer was achieved, since the fibril was not stretched out of position, although the minimal contact led to a later detachment. The detachment is also more pronounced towards the sides, where less contact between filaments had been predicted. In some sections of the scaffolds made using the “constant radius” strategy with radii of 17.064 mm and 14 mm, the filament being deposited became straight instead of maintaining the curvature, indicating no contact with the previously deposited material. For the 17.064 mm radius scaffolds, this problem is also more noticeable towards the sides. On the other hand, for the 14 mm radius scaffolds, due to the proximity of the fibrils being printed towards the sides, they were printed as a surface instead of individual filaments. Consequently, for those scaffolds, the detachment was seen not at the extremity but closer to the center of the scaffold.

**Figure 9:**
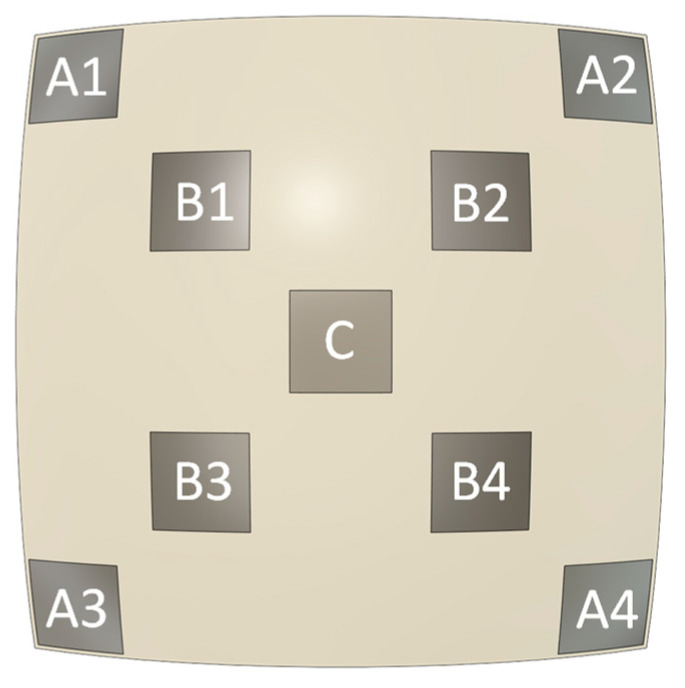
Scaffold sections (VOIs) identification of where a particular shape analysis is dedicated.

As represented in Figure 9, scaffold characterization focused on specific volumes of interest (VOIs) to analyze local differences in printed scaffolds due to the different scaffold structures resulting from the two design strategies and radius variation. Properties of regions in the CAD models, approximately corresponding to these sections, were calculated for comparison.

The scaffold properties were estimated from μ-CT reconstructions and are presented in Table 2 and Supplementary Table S1. Measurements of porosity (%), interconnectivity (%), and surface area/volume ratio were made for the sections of the scaffolds. The experimentally estimated values for porosity are frequently slightly higher than the ones determined from the CAD models (Table 2), which could indicate an under-extrusion of filament during printing. 

Different results for the porosity variation, from the extremities of the scaffolds towards the center, are expected according to the design strategy. In the “concentric radius” strategy, due to the enlargement towards the extremities, porosity was generally higher at the extremities and lower towards the center, as expected. Additionally, the variation was greater in the scaffold with the smaller radius. On the contrary, in the “constant radius” strategy, porosity was lower in the corners and increased towards the center of the scaffolds. The variation was also more pronounced in the scaffolds with the smaller radius. These results are expected, as scaffolds with larger radius will approximate an orthogonal scaffold with equivalent dimensions. 

Concerning the surface area/volume ratio (Supplementary Table S1), filaments are being deposited in single strands over each other, so no expressive variations should be expected between all sections of the scaffolds. The observed ratio ranged from 13.6 mm^−1^ to 16.6 mm^−1^, which can be attributable to experimental variation and differences in the VOIs considered. Regarding interconnectivity, for every section considered, the value is approximately 100% (Supplementary Table S1), meaning there were no occlusions in the pores resulting from the manufacturing procedure.

### 3.3. Mechanical Behavior of Curved Scaffolds under Compressive Loading

As explained in Section 3.1.3, dedicated assemblies were designed and printed for mechanical assessment. Such assemblies integrate the different curved scaffolds designed into the middle of solid blocks placed on both sides of the scaffolds, allowing the compression of curved scaffolds between the two flat plates of the test machine in spite of their curvature. Specimen pictures of these assemblies, before and after compressive tests, are shown in Supplementary Figure S4. The obtained stress–strain curves and corresponding compressive moduli, calculated from the initial linear regions, are shown in Supplementary Figure S5 and Table S2, respectively. Since the solid block has a compact structure, it displays a higher modulus. Upon the insertion of the porous scaffolds into the blocks, the calculated modulus decreases, pointing to a reduction in resistance to deformation.

The calculation of the stresses in Supplementary Figure S5 was performed with the force being applied considering two distinct areas: (i) the cross-sectional area, corresponding to the area of contact of the machine with the blocks; (ii) the area of the curved surfaces that define the curvature of the scaffolds. Considering the conventional cross-sectional area, statistically significant differences in the compressive modulus were only found among the assemblies when compared to the concentric 20 mm insertion, but these differences can result from wider dispersion of the experimental results. The curved surface area can be considered as more representative of the stress distribution on the assemblies. In this case, the force is considered to be applied over a larger area, resulting in the calculation of lower moduli for scaffolds with greater curvatures but also increasing the significance differences between the estimated moduli.

Concerning the yield stress, calculated by the 0.2% offset method, a similar behavior to the compressive modulus was observed, with the insertion of a porous structure within the block leading to a significant reduction in the yield stress in comparison to the solid block (Supplementary Figure S5 and Table S2). Again, when considering the stress calculated in relation to the curved surface areas instead of the cross-sectional areas, the determined yield stress was reduced, and the differences between the stresses depending on the curvatures became more significant (Supplementary Figure S6).

**Figure 10:**
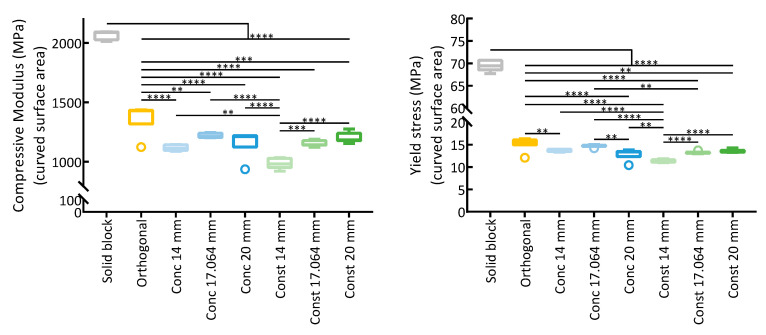
Compressive moduli (determined from initial linear regions) and yield stress (calculated with the 0.2% offset method) for the mechanical compression of the scaffold and block assemblies, considering the area of the curved surface of the scaffolds for the calculation of stresses (n = 7; ** *p* < 0.01, *** *p* < 0.001, **** *p* < 0.0001).

Furthermore, two linear regions are observed in the stress–strain plots, suggesting different stages in the compression. In an initial stage, the strain would be mostly absorbed by the scaffold insertions, and due to its small width, the onset of plasticity is observed earlier. A second linear region can be observed, corresponding to the compression of a structure where the scaffold inserts have collapsed. This behavior shows some resemblance to the solid structure. Note that while the moduli determined with the assemblies should not correspond to the moduli of the scaffolds, since some deformation might also have occurred in the solid blocks, the results obtained nevertheless allow making a comparison between the assemblies. In Figure 10, the moduli and yield strength calculated considering the curved surface area of the scaffolds are shown.

### 3.4. Finite Element Analysis

A deeper understanding of the compressive behavior of the assemblies can be gathered from finite element analysis. Supplementary Figure S7 shows the von Mises stresses calculated in the compression of the scaffold and block assemblies. The existence of two planes of symmetry allowed the simulation to be run in a fraction of the assembly. Only this fraction, corresponding to a quarter of the assembly, is represented in the figures.

For all assemblies, the simulations show higher von Mises stresses in the scaffold fibrils perpendicular to the direction of the applied load. On the other hand, the stresses in the curved fibrils, closer to a parallel orientation in relation to the applied load, are much smaller. It is understandable that higher stress is obtained upon a force being transmitted across smaller material areas, as occurs in the transition of the solid blocks to the scaffold sections in the assemblies. Additionally, higher stresses are associated with more extensive deformations. Therefore, when compressing the assemblies, a more extensive deformation is observed in the scaffold region, in comparison to the solid blocks of the assemblies. Still, the deformation is not limited to the scaffold region, and some deformation is also predicted in both solid blocks adjacent to the scaffolds, although not as noticeable as that in the scaffold sections (Supplementary Figure S8). Comparing the assemblies and the homogeneous solid block, the range of stress variation is smaller in the compression of the solid block. Since there is no sudden transition from blocks to scaffolds, the stress in the solid block does not reach values as high as in the assemblies. Furthermore, since in the assemblies the stress is mostly located within the scaffold region, the stress is not as high in the assembly block region as in the corresponding region of the solid block.

**Figure 11:**
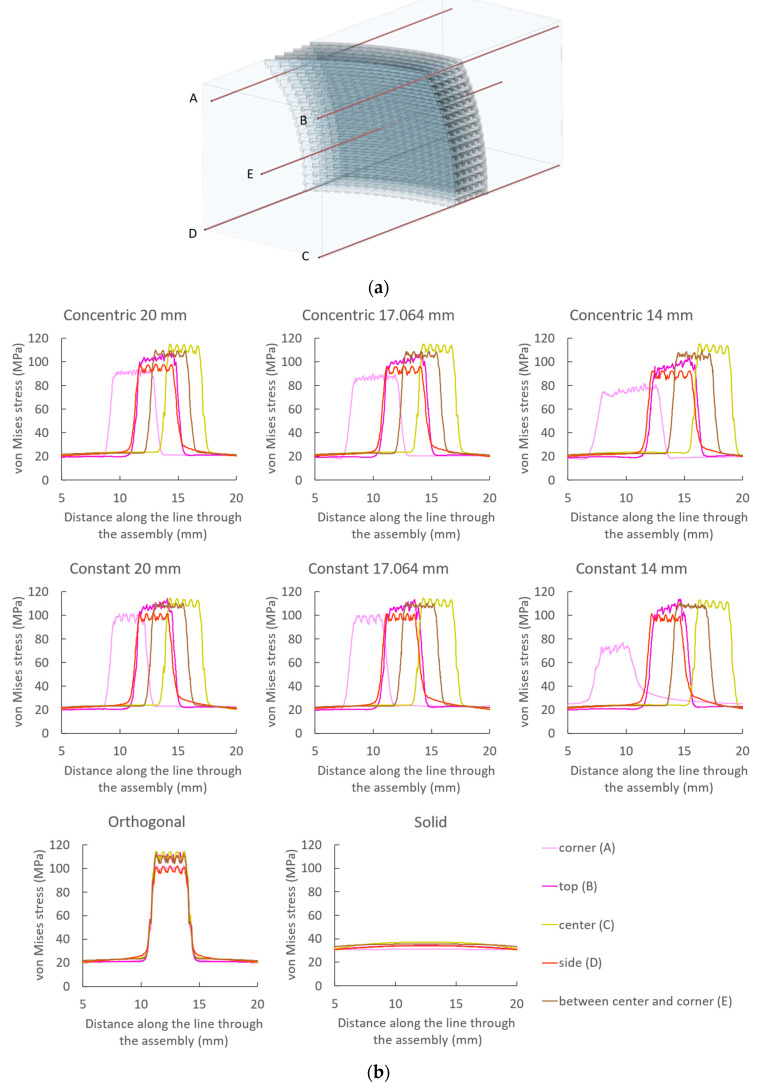
Von Mises stresses in the compression of scaffolds and block assemblies: (**a**) Position of the lines (red segments) along which stresses were determined. (**b**) Variation in stress along the lines represented in (**a**) corresponding to five different locations in the assemblies (corner (A), top (B), center (C), side (D) and between center and corner (E)).

To assess the effect of the curvature and the design strategies on the estimated von Mises stresses, a stress plot was obtained through the assemblies and the solid block in five distinct locations, represented by the red segments in Figure 11a. Those locations are in the following regions: corner (A), top central (B), center (C), side middle (D), and midway between corner and center (E). Figure 11b shows the calculated von Mises stresses. An average of the von Mises stress was calculated on the scaffold region of the assemblies and in the corresponding section in the middle of the solid block (Supplementary Figure S9). Compared to the assemblies, the calculated average von Mises stress in the solid block is always inferior, reflecting the load distribution by a larger area. For the assemblies, a pattern is observed for the calculated stresses according to their location. Indeed, these stresses are higher in the center and become smaller towards the edges of the scaffold region of the assemblies. This reduction in estimated stresses is more evident towards the corners and the sides than towards the top positions. This is an observation that we hypothesize to be due to the scaffold fibrils’ orientation on the corners and on the sides that may allow for a greater freedom for deformation and, therefore, a reduction of stress. 

Additionally, with an increase in curvature, a greater freedom for deformation could also be anticipated, so an overall slight decrease in stress might be calculated. Moreover, the variation in stress in the different positions can also be analyzed relating to the curvature. On the orthogonal scaffold insert, the calculated stresses are smaller towards the edges. This observation is more marked for curved scaffolds, with smaller stresses estimated as the curvature increases. Further comparison of stresses in the same region of assemblies with different curvatures can provide further insights. In the center of the scaffolds, due to their geometric resemblance at these locations, the values of stresses estimated are very similar, regardless of their design. However, the reduction in stress values becomes more noticeable farther from the center, with the greater variations observed in the corners. The reduction is also more noticeable in the scaffolds manufactured with the “concentric radius” strategy than with the “constant radius” strategy, which could be a result of the structural widening towards the corners allowing a greater freedom for deformation in the former strategy (Figure 11b and Figure S9).

## 4. Discussion

The ability to recreate the precise shape and heterogeneous architecture of tissues in the human body remains a challenge in TE strategies. The purpose could be, for instance, to create scaffolds fitting specific injured regions, inevitably variable by nature, and following the contours of surrounding tissue to achieve an appearance close to the native one. Additionally, at a smaller scale, recreating branching patterns of tissues, accounting for the possibility of vascularization, and gradient features would also be of great interest to produce more robust tissue substitutes [59,60]. In this sense, available medical imaging techniques, such as computed tomography and magnetic resonance imaging, have been combined with CAD software-assisted design and additive manufacturing methods to fabricate “patient-tailored” scaffolds able to fit perfectly into the defect [25]. Despite the recent shift in the TE field towards more complex scaffold designs, the produced constructs still fail to fully recreate the complexity of the native tissues and the curvature concept has been highly overlooked. In this work, we present a novel method to reproduce the curvature of the knee OC tissue, using a sphere as an approximation and as a template for the design and construction of scaffolds. Using an AM technique for scaffold manufacturing, the developed design presents the advantage of being highly reproducible. Furthermore, the use of parametric design allowed precise definition of scaffold parameters relating to curvature and overall dimensions, which we demonstrated could be altered to desired arbitrary values by modelling with three distinct curvatures. Thus, the purpose of having a scaffold that could fulfil specific curvature needs was achieved.

Fibers of material are designed at a specific distance from each other to manufacture, by extrusion, scaffolds exhibiting a porous structure. While more pronounced curvatures are expected for a lower design radius used, there is a minimal limit of the radius value that can be utilized without the use of supports. Still, the use of supports should be avoided due to constraints on printing fine details and potential scar formation upon support removal [61]. Therefore, with the introduction of a curvature, a procedure to evaluate the printability without using supports was devised, and the lower limit radius that allows printability without supports calculated by this procedure was 17.064 mm, considering a scaffold with 20.1 mm × 20.1 mm dimensions. Having in mind the objective of achieving an OC construct, it is worth highlighting that such a curvature is superior to the one found in the native tissue [62,63,64], and thus, these designs and manufacturing techniques can easily be adapted to the creation of clinically relevant scaffolds.

Considering the results obtained from the SEM and μ-CT imaging of the manufactured scaffolds (Figure 7 and Figure 8), the printability limit is in accordance with what was expected. Clear deficiencies are observed when the scaffold radius of curvature is lower than this limit, and prints faithful to the design are observed when the radius is higher than it. Some faults observed in the prints made near the limiting radius of curvature can be considered reasonable to occur, as room for depositing new layers over the existing material is close to the physical limit. Being closer to the limit of printability also resulted in an increase in stringing printing artifacts. This suggests that the smaller support at the extremities of the scaffolds, where those artifacts were predominately observed, resulted from the release of the residual pressure at the nozzle, which compromised printability performance and potentiated appearance of those artifacts. A better tuning of the nozzle retraction settings could be attempted. However, it would not affect the lack of support at the steepest regions of the scaffolds with higher curvature. The fidelity potential of the selected manufacturing process can also be verified in the porosity measurements obtained from the μ-CT imaging (Table 2). These measurements confirm that, from the center of the scaffold towards the corners, the porosity decreases for the “constant radius” and increases for the “concentric radius” design strategies. Furthermore, as expected, this variation was more evident in the scaffolds with higher curvature. A valuable feature, quantified by μ-CT, was a pore interconnectivity of 100%, which is favorable for cell migration and colonization throughout the entire structure and also for widespread nutrient supply and waste removal [65]. Previous studies have shown that cell fate is highly affected by pore size [57,66]. Fibrils were designed with a width of 300 μm and, at the center of the scaffold, placed at 300 μm from each other. Such distance defines the pore size, which would increase or decrease towards the corners, whether built according to the “concentric radius” or “constant radius” strategies, respectively. Although the literature shows some variability, it has been suggested that larger pores (above 250–300 μm) favor a differentiation towards the osteogenic lineage by allowing vascularization to occur. Conversely, with smaller pores, blood vessel formation is hindered, and an environment closer to cartilage tissue is created, so chondrogenic differentiation is favored [67,68]. In the case of chondrogenesis, further differences in cell behavior have been reported, with smaller pores promoting type II collagen and aggrecan production, while larger pores seem more favorable to cell proliferation [67]. Since the design is automated in this work, it would be feasible to change parameters so that the differentiation would be favorable towards any specific lineage. Considering this, our future work will comprise the evaluation of the biological performance of these curved scaffolds through the study of their effects on the proliferation and differentiation of human mesenchymal stem/stromal cells, in comparison to standard planar orthogonal scaffolds. 

To perform the compressive testing of the produced scaffolds, an assembly with solid blocks of material had to be conceived so that a load could be applied to the flat surfaces of the testing machine. However, being constituted by the same material as the scaffold, it is not possible to exclude the compression in the blocks. Still, a clear reduction in the yield strength is observed when scaffold inserts are introduced inside a solid block, as is a reduction in the compressive modulus, pointing to an initial compression exerted mainly in the scaffold region (Supplementary Figure S5). Compared to the standard orthogonal scaffold design, the introduction of curvature resulted in a small decrease in yield strength (considering the curved surface areas for the calculation), more noticeable with greater curvatures but still maintaining a similar magnitude (Supplementary Figure S6).

Considering only the orthogonal scaffold design, a compression test was also performed with just the scaffold insert (Supplementary Table S3 and Figure S10). Compared to the assembly with blocks, the yield strength is slightly lower (11.6 MPa vs. 15.2 MPa), which might indicate that some of the force being applied to assemblies is causing deformation of the blocks. The determined modulus for the scaffold alone was 215 MPa, which is in accordance with values reported by other works using the same PLA material [69]. Acknowledging the aims of TE, for optimal scaffold integration, their ideal properties should match the ones of the tissue they are intended to replace. Therefore, these scaffolds are closer to the strength and modulus of trabecular bone [70], rather than cartilage (lower modulus and strength) [47] or cortical bone (higher modulus and strength) [71]. In this regard, other materials and porosity variations could be envisaged to approximate the mechanical properties of those tissues. Moreover, the present study only evaluated the mechanical behavior of the scaffolds under specific conditions, and therefore, further work will include the analysis of their performance under more varied and realistic conditions (e.g., cyclic loading).

Taking advantage of the capabilities of numerical simulations, it was possible to better understand the assemblies’ behavior during compression, improving on what was reached experimentally. The material was considered only as linear elastic, and the compression was restricted to the elasticity limit strain observed experimentally, so that the prescribed compression in the simulation would be within legitimate limits. The used modulus for the FEA simulations was obtained from the compression of the solid block (2062 MPa, Table S2). This value is within the range of the ones found in the literature for the compression of PLA specimens manufactured by FFF [72,73], and considering that the block was produced with the same manufacturing conditions as the assemblies prepared for the scaffolds mechanical tests, the choice of this value is in our opinion reasonable. Note that compression modulus value estimation depends on several factors, including testing mode (e.g., tension or compression), material specimens’ preparation method (e.g., injection molding, FDM), and parameters used (temperature, extrusion speed, and infill pattern) [74,75]. Based on the results for von Mises stresses (Figure 11 and Supplementary Figure S8), it is possible to confirm that the load is mostly absorbed by the fibrils aligned with the applied force, yet some deformation is observed in the adjacent solid blocks. This result supports the hypothesis that the previously determined experimental modulus is not exclusively due to the scaffold compression. The simulation also reveals how the load is transmitted, which is a consequence of the scaffold design. The straight fibrils aligned with the applied load accommodate most of the load, and very little is transmitted to the curved fibrils. In order that the scaffolds could have a curved porous structure and be manufactured by FFF, the fibrils needed to be in the designed orientation. Therefore, the curved structure resulted in a limitation to the freedom of design, with implications for the transmission of loads. Additional designs can be proposed with the perspective of a more widespread distribution of loads, and numerical simulations can be employed for a fast evaluation of the mechanical behavior of such designs.

## 5. Conclusions

In the present study, we presented a novel strategy for designing and manufacturing curvature-featuring scaffolds using a mathematical approach to precisely define their structure. With the variation in design parameters, different curvature-featuring scaffolds could easily be obtained. In the context of scaffolds for OC TE, the ability to manufacture scaffolds that mimic the natural tissue and are adaptable to injury site structure variability has the potential to achieve better integration and successful therapeutic outcomes. A procedure was conceived to determine the manufacturability of the scaffolds, depending on their radius and curvature, which SEM and μ-CT experimental imaging corroborated. Mechanically, the curved scaffolds showed comparable properties to the common orthogonal scaffolds. A greater insight into their mechanical behavior was obtained from FEA, identifying the areas subjected to greater stresses. The insight provided by those models highlighted the potential of conjugating numerical modelling with experimental data towards the development of improved scaffolds for TE strategies.

## Figures and Tables

**Table 1:** Summary of dimensions of curved scaffolds and solution to the limit printability condition.

Scaffold Dimension	Assigned Parameters	Calculated Limit Curvature
height = 20.1 mm width = 20.1 mm	*L* = 10.05 mm *h_dist* = 0.3 mm *v_dist* = 0.3 mm	*R_side of scaffold_* = 13.790 mm *α* = 44.12° *β* = 45.88° *R* = 17.064 mm

**Table 2:** Percentage (%) of porosity of the curved scaffold CAD models and of the printed scaffolds, obtained from the μ-CT analysis. The sections (VOIs) of the scaffolds are indicated in Figure 9.

VOI	Concentric						Constant						Orthogonal	
	14 mm		17.064 mm		20 mm		14 mm		17.064 mm		20 mm			
	CAD	μ-CT	CAD	μ-CT	CAD	μ-CT	CAD	μ-CT	CAD	μ-CT	CAD	μ-CT	CAD	μ-CT
A1	60.3	64.2	59.1	64.0	58.4	56.9	45.1	48.7	52.0	53.7	53.7	50.8	56.3	62.3
A2		70.8		63.4		61.6		52.9		55.7		53.9		
A3		67.3		64.6		61.6		45.0		53.9		62.5		
A4		67.0		66.4		61.3		52.9		56.8		53.9		
C	56.3	62.1	56.3	65.9	56.3	64.7	56.3	65.0	56.3	61.0	56.3	60.3		
B1	57.6	63.7	57.1	64.3	57.0	66.2	55.7	58.7	55.8	58.7	56.0	63.4		
B2		65.3		64.9		56.4		61.0		58.4		63.9		
B3		63.5		66.2		65.4		57.5		61.8		62.4		
B4		62.1		61.9		65.1		54.9		59.0		59.5		

## Data Availability

The data that support the findings of this study are available from the corresponding authors upon proper request.

## Supplementary Materials

The following supporting information can be downloaded at https://www.mdpi.com/article/10.3390/polym15092129/s1. Figure S1: CAD models of the scaffold and block assemblies; Figure S2: Schematic representation of the scaffold and blocks assemblies imported into COMSOL software; Figure S3: Designed orthogonal scaffold with the same side lengths and top projected as the curved scaffolds; Figure S4: Representative images of the scaffold and blocks assemblies before (top) and after (bottom) the mechanical compression test; Figure S5: Stress-strain curves of the mechanical compression of the block and scaffold assemblies and respective compressive moduli values; Figure S6: Yield stress in the compression of the scaffold and blocks assemblies calculated with the 0.2% offset method from the stress-strain curves, in relation to the cross-sectional area (top) and the area of the curved surface of the scaffolds (bottom); Figure S7: Von Mises stresses in the numerical finite element analysis (FEA) of the mechanical behavior in compression of the block and scaffold assemblies; and a solid block with the same dimensions; Figure S8: Numerical simulation results of the rate of deformation of the scaffold and block assemblies along the direction of compression; Figure S9: Average von Mises stresses in the scaffold section of the assemblies and for an equivalent section of the solid block calculated from the plots in Figure 11; Figure S10: Stress-strain curves of the mechanical compression of the orthogonal scaffold without adjacent blocks; Table S1: Structural features (interconnectivity and surface area/volume ratio) of sections of the scaffolds measured from the μ-CT analysis; Table S2: Compressive modulus and yield stress (obtained with the 0.2% offset method) calculated for the solid block and for the scaffold and block assemblies; Table S3: Compressive modulus and yield stress (obtained with the 0.2% offset method) calculated for the orthogonal scaffold without adjacent solid blocks.
