# Supplementary material for: A Novel Approach for Design and Manufacturing of Curvature-Featuring Scaffolds for Osteochondral Repair
Source: Polymers (Basel). 2023 Apr 29;15(9):2129. doi: 10.3390/polym15092129 (PMC10181173; doi:10.3390/polym15092129)
Supplement: Supplementary file 1 [file polymers-15-02129-s001.zip › polymers-2329649-supplementary.pdf]

Supplementary materials

A novel approach for design and manufacturing of curvature-featuring scaffolds for osteochondral repair

Pedro Marcelino [1,2,3], João Carlos Silva [1,2,3*], Carla Moura [3,4,5], João Meneses [3], Rachel Cordeiro [3,6], Nuno Alves [3,4,7+], Paula Pascoal-Faria [3,4,8*+], Frederico Castelo Ferreira [1,2*+]

[1] Department of Bioengineering and iBB-Institute for Bioengineering and Biosciences, Instituto Superior Técnico, Universidade de Lisboa, Av. Rovisco Pais, 1049-001 Lisboa, Portugal

[2] Associate Laboratory i4HB – Institute for Health and Bioeconomy, Instituto Superior Técnico, Universidade de Lisboa, Av. Rovisco Pais, 1049-001 Lisboa, Portugal

[3] CDRSP-Centre for Rapid and Sustainable Product Development, Polytechnic of Leiria, Rua de Portugal-Zona Industrial, 2430-028 Marinha Grande, Portugal

[4] Associate Laboratory for Advanced Production and Intelligent Systems (ARISE), Porto, Portugal

[5] Polytechnic Institute of Coimbra, Applied Research Institute, Rua da Misericórdia, Lagar dos Cortiços – S. Martinho do Bispo, 3045-093 Coimbra, Portugal

[6] Veterinary Clinics Department, Abel Salazar Biomedical Sciences Institute, University of Porto, Rua de Jorge Viterbo Ferreira 228, 4050-313 Porto, Portugal

[7] Department of Mechanical Engineering, School of Technology and Management, Polytechnic of Leiria, Morro do Lena – Alto do Vieiro, Apartado 4163, 2411-901 Leiria, Portugal

[8] Department of Mathematics, School of Technology and Management, Polytechnic of Leiria, Morro do Lena – Alto do Vieiro, Apartado 4163, 2411-901 Leiria, Portugal

[*] Correspondence: joao.f.da.silva@tecnico.ulisboa.pt (JCS); paula.faria@ipleiria.pt (PPF); frederico.ferreira@tecnico.ulisboa.pt (FCF)

[+] Joint last authors

Contents

A. Assemblies of scaffolds with solid blocks for the mechanical compressive testing 2

A1. CAD models of the scaffold and blocks assemblies 2

A2. Illustration of the geometry of CAD models imported into COMSOL and solid mechanics constrains applied to their surfaces 4

A3. Orthogonal scaffold model 4

B. Characterization of the scaffold and blocks assemblies 5

B1. Curved scaffold characterization and shape fidelity analysis 5

B2. Representative pictures of scaffold and blocks assemblies before and after compression5

B3. Mechanical characterization of scaffold and blocks assemblies 6

B4. Finite Element analysis (FEA) of the compression of assemblies 8

B5. Mechanical characterization of the orthogonal scaffold 11

A. Assemblies of scaffolds with solid blocks for the mechanical compressive testing

A1. CAD models of the scaffold and blocks assemblies

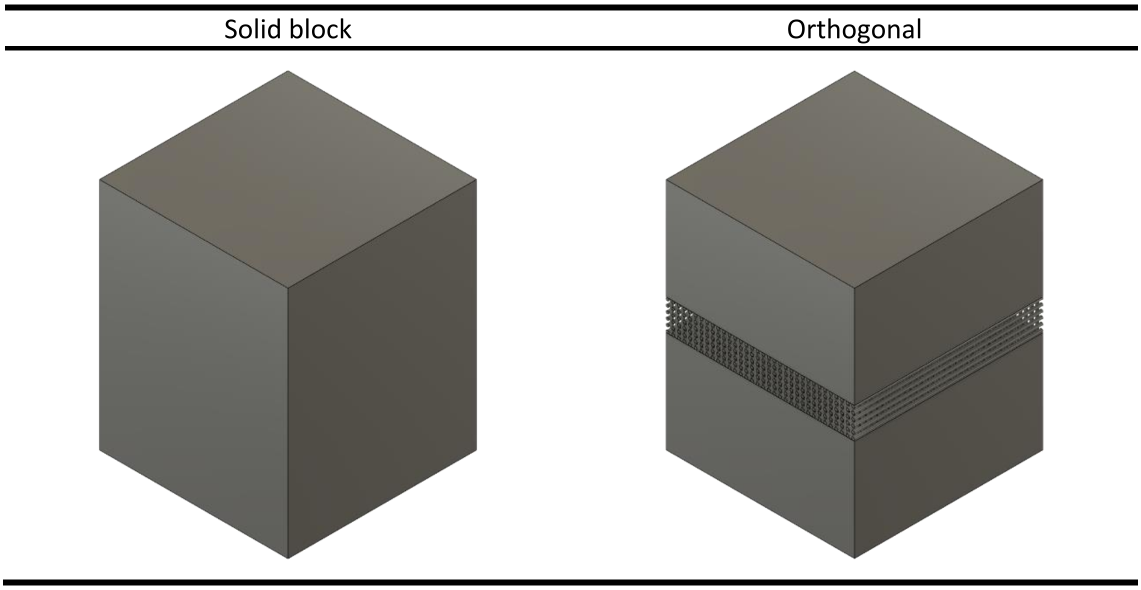

Figure S1.1 *CAD models of the scaffold and blocks assemblies (solid block and orthogonal scaffold), which were designed and 3D printed for the mechanical compressive testing. For all assemblies the global dimensions are the same (25 mm height and 20.1 mm × 20.1 mm top projected area).*

Constant radius	Concentric radius
14 mm	14 mm
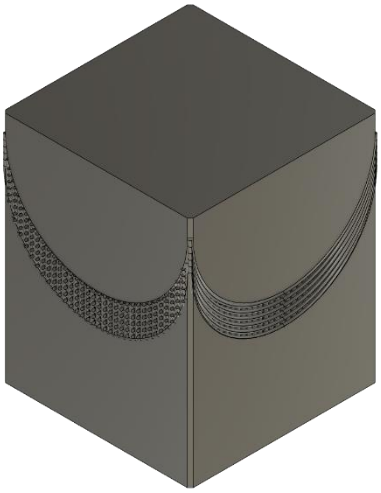	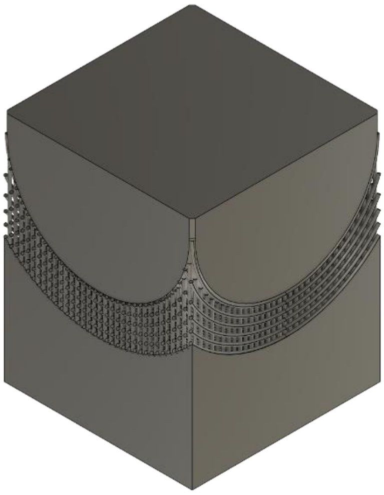
17.064 mm	17.064 mm
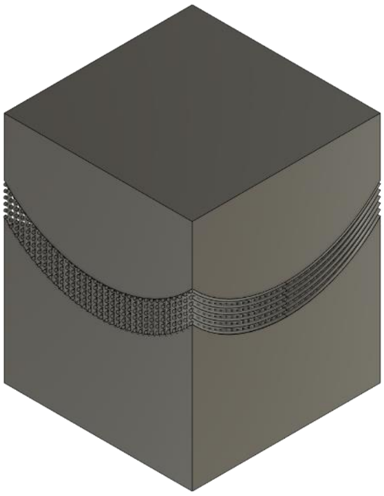	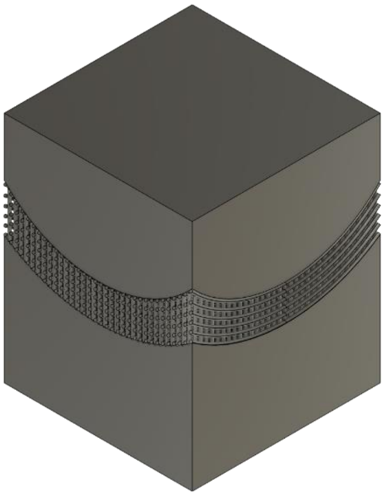
20 mm	20 mm
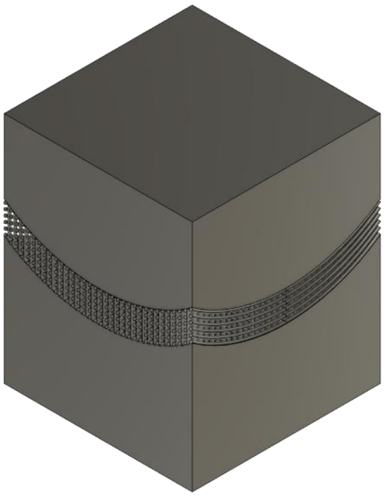	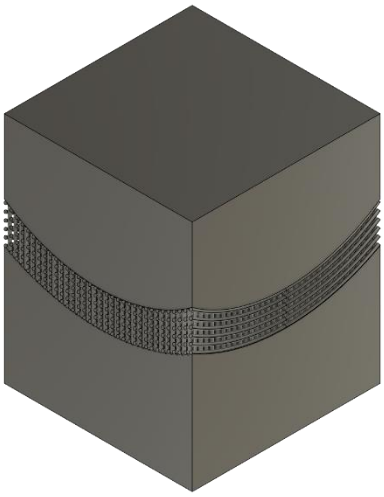

Figure S1.2. *CAD models of the scaffold and blocks assemblies ("constant radius" and "concentric radius"), which were designed and 3D printed for the mechanical compressive testing. For all assemblies the global dimensions are the same (25 mm height and 20.1 mm × 20.1 mm top projected area).*

A2. Illustration of the geometry of CAD models imported into COMSOL and solid mechanics constrains applied to their surfaces

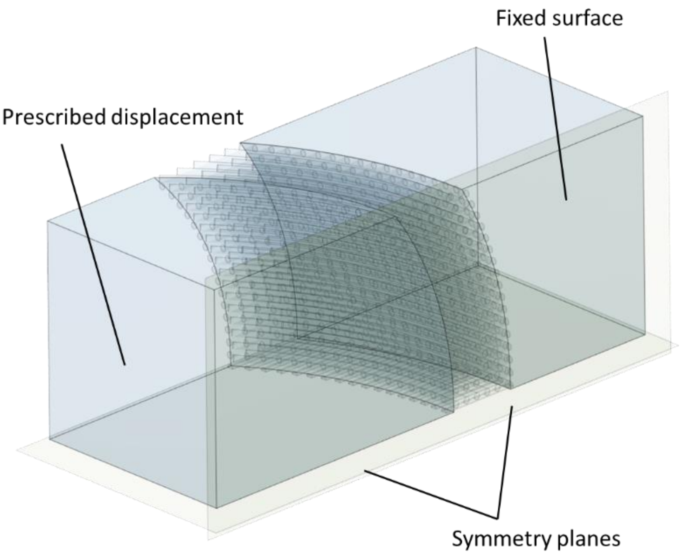

Figure S2. *Schematic representation of the scaffold and blocks assemblies imported into COMSOL. The indicated symmetry planes were defined to simplify the numerical computation. The planar faces defined as fixed surface and to which a displacement was prescribed are also indicated.*

A3. Orthogonal scaffold model

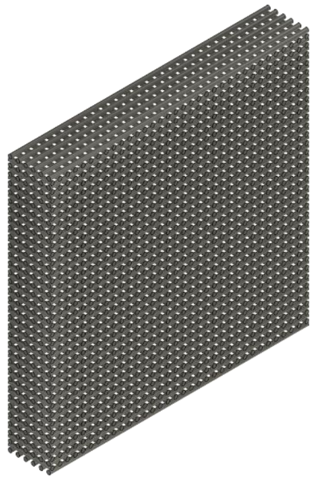

Figure S3. *Designed orthogonal scaffold with the same side lengths and top projected as the curved scaffolds.*

B. Characterization of the scaffold and blocks assemblies

B1. Curved scaffold characterization and shape fidelity analysis

Table S1. *Structural features (interconnectivity and surface area/volume ratio) of sections of the scaffolds measured from the µ-CT analysis.*

VOI	Concentric						Constant					
	20 mm		17.064 mm		14 mm		20 mm		17.064 mm		14 mm	
	IC %	SA/V mm^{-1}	IC %	SA/V mm^{-1}	IC %	SA/V mm^{-1}	IC %	SA/V mm^{-1}	IC %	SA/V mm^{-1}	IC %	SA/V mm^{-1}
A1	100	14.3	100	15.4	100	15.7	100	14.1	100	15.8	100	14.4
A2	100	15.2	100	14.6	100	17.1	100	14.0	100	15.6	100	14.3
A3	100	14.6	100	14.8	100	15.3	100	16.2	100	15.0	100	11.9
A4	100	14.5	100	15.3	100	15.2	100	15.0	100	14.7	100	13.9
C	100	15.6	100	15.8	100	15.4	100	15.2	100	23.4	100	16.6
B1	100	16.4	100	15.0	100	15.5	100	16.6	100	20.8	100	15.2
B2	100	13.9	100	15.9	100	16.0	100	16.0	100	20.9	100	15.5
B3	100	15.4	100	15.9	100	14.8	100	15.6	100	19.8	100	14.1
B4	100	15.5	100	14.4	100	15.2	100	15.4	100	18.8	100	13.8

B2. Representative pictures of scaffold and blocks assemblies before and after compression

Solid	Orthogonal	Concentric (mm)			Constant (mm)		
		20	17.064	14	20	17.064	14

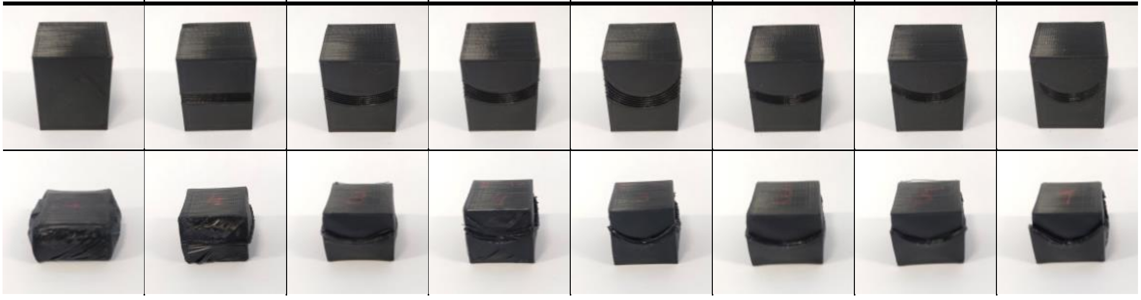

Figure S4. *Representative images of the scaffold and blocks assemblies before (top) and after (bottom) the mechanical compression test.*

B3. Mechanical characterization of scaffold and blocks assemblies

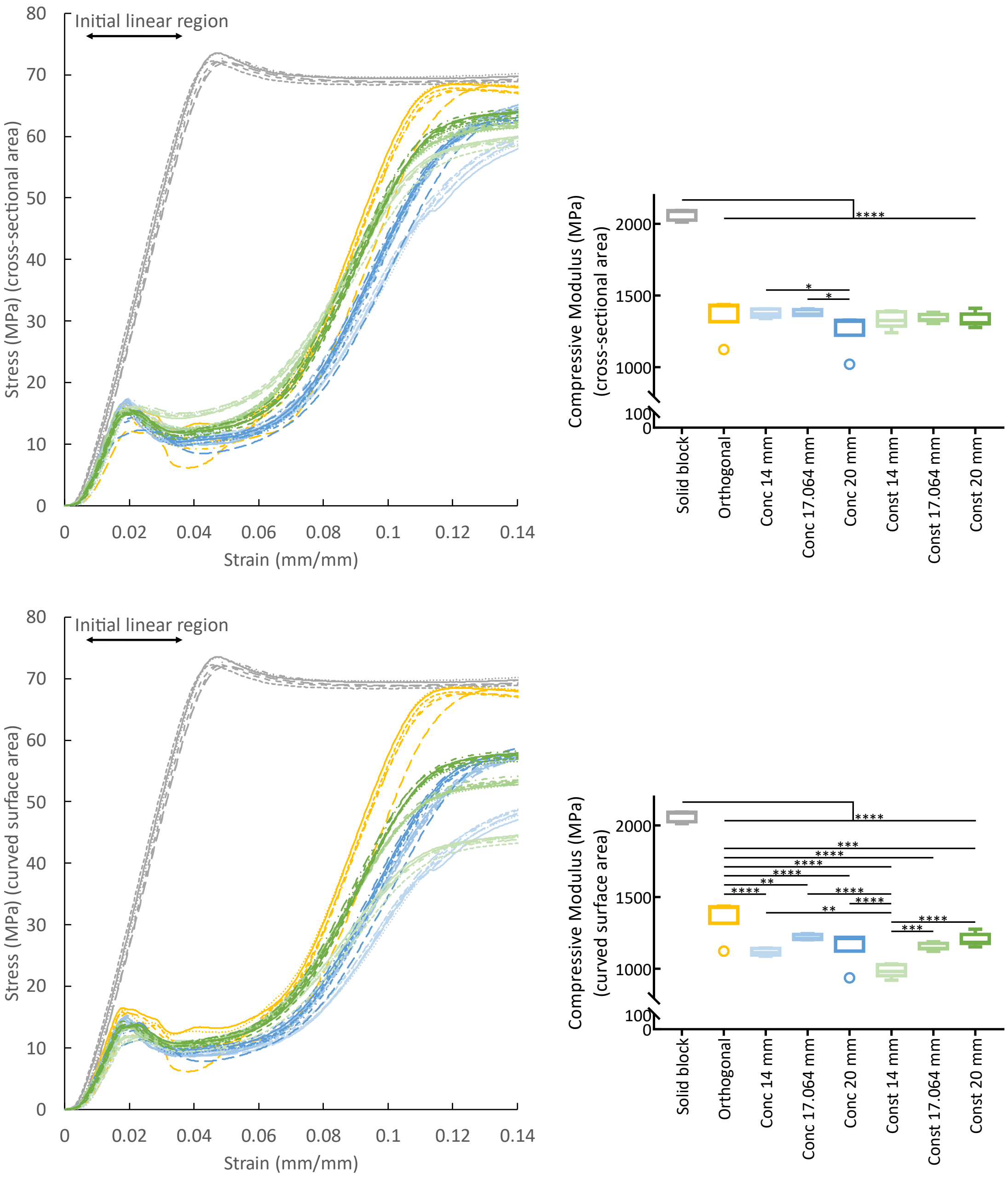

Figure S5. *Stress-strain curves of the mechanical compression of the block and scaffold assemblies and compressive moduli determined from the initial linear regions, considering the cross-sectional area (top) and the area of the curved surface of the scaffolds (bottom) (n=7).*

Table S2. *Compressive modulus and yield stress (obtained with the 0.2% offset method) calculated for the solid block and for the scaffold and block assemblies (n=7).*

Solid	Orthogonal	Concentric (mm)			Constant (mm)		
		20	17.064	14	20	17.064	14
Compressive modulus (MPa) (cross-sectional area)							
2062±35	1357±115	1376±27	1377±19	1257±112	1326±57	1340±26	1327±46
Compressive modulus (MPa) (curved surface area)							
-	-	1117±22	1218±17	1154±103	982±42	1151±23	1199±42
Yield stress (MPa) (cross-sectional area)							
69.4±1.1	15.2±1.4	16.9±0.3	16.6±0.3	14.0±1.3	15.4±0.4	15.4±0.3	15.0±0.4
Yield stress (MPa) (curved surface area)							
-	-	13.7±0.3	14.7±0.3	12.8±1.2	11.4±0.3	13.2±0.3	13.6±0.3

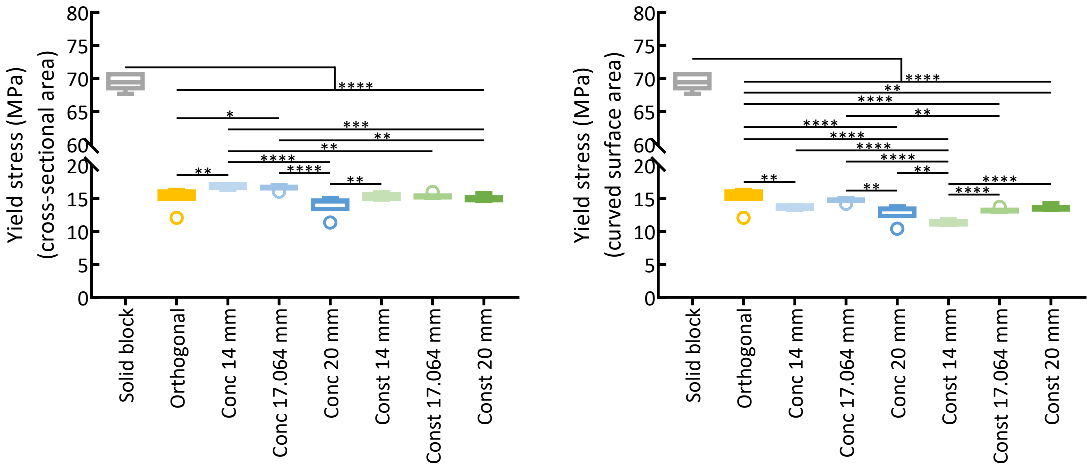

Figure S6. *Yield stress in the compression of the scaffold and blocks assemblies calculated with the 0.2% offset method from the stress-strain curves, in relation to the cross-sectional area (top) and the area of the curved surface of the scaffolds (bottom) (n=7).*

B4. Finite Element analysis (FEA) of the compression of assemblies

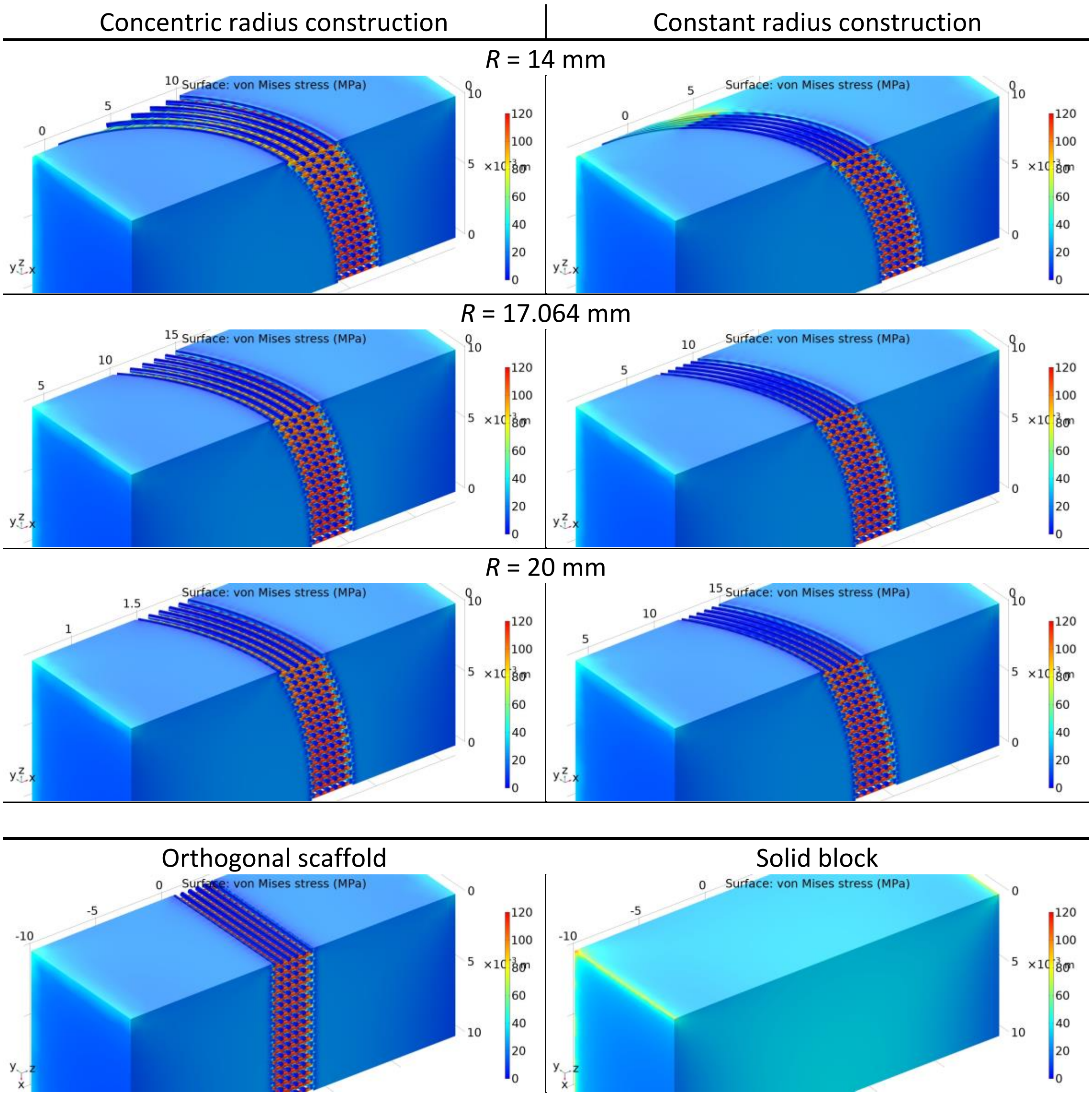

Figure S7. *Von Mises stresses in the numerical finite element analysis (FEA) of the mechanical behavior in compression of the block and scaffold assemblies; and a solid block with the same dimensions.*

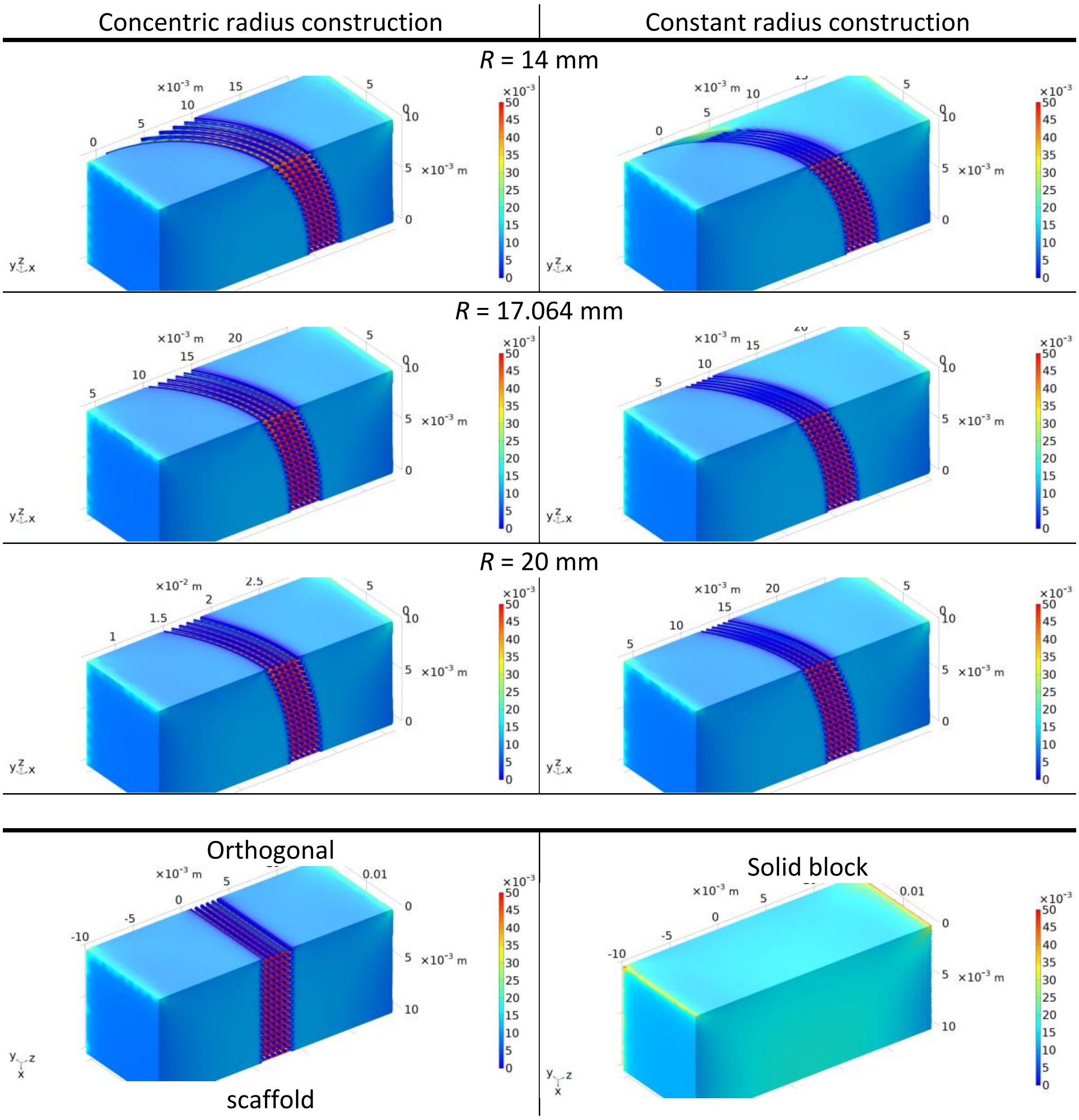

Figure S8. *Numerical simulation results of the rate of deformation of the scaffold and block assemblies along the direction of compression.*

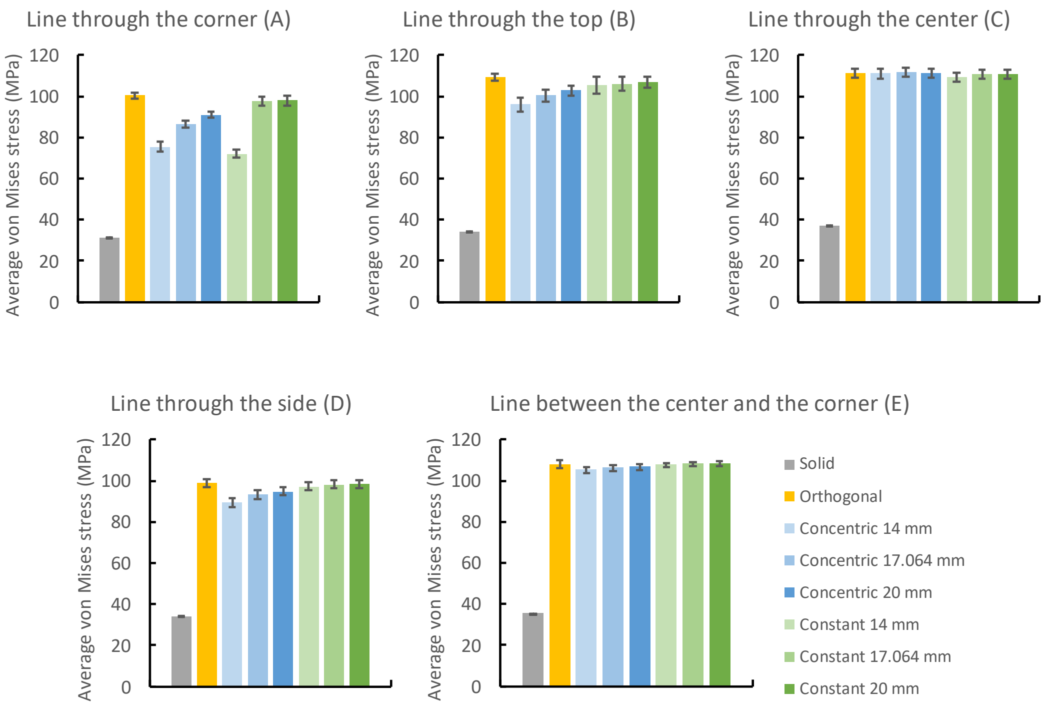

Figure S9. *Average von Mises stresses in the scaffold section of the assemblies and for an equivalent section of the solid block calculated from the plots in Figure 11 (error bars denote the standard deviation of the plotted values).*

B5. Mechanical characterization of the orthogonal scaffold

Table S3. *Compressive modulus and yield stress (obtained with the 0.2% offset method) calculated for the orthogonal scaffold without adjacent solid blocks (n=7).*

Compressive modulus (MPa)	Yield stress (MPa)
215±10	11.6±0.7

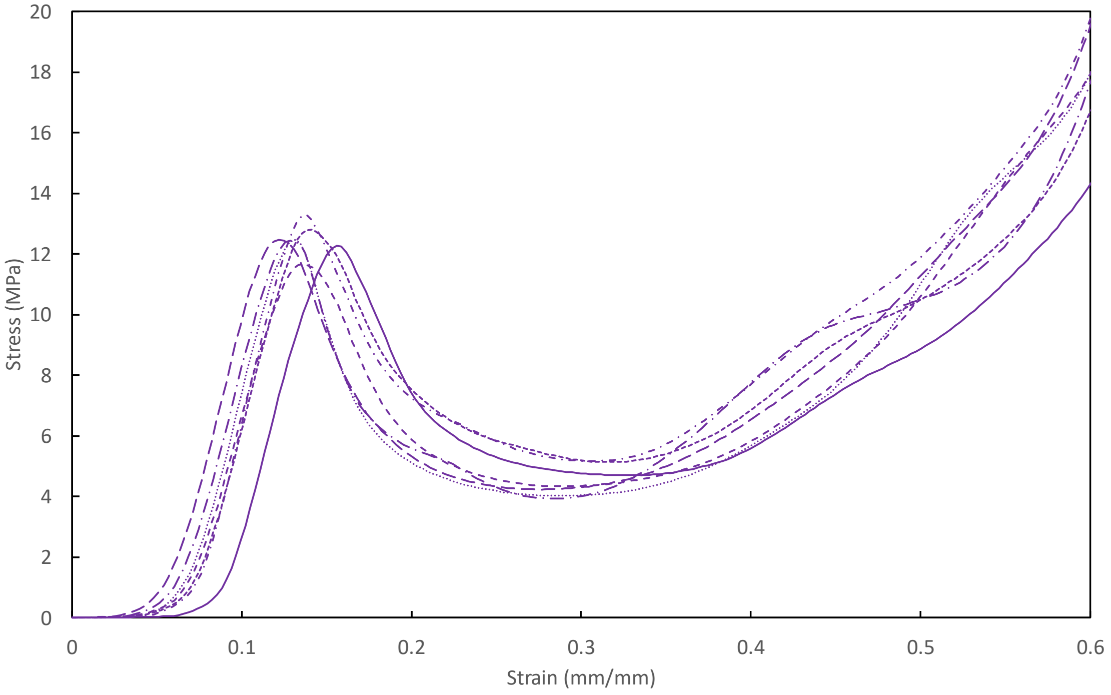

Figure S10. *Stress-strain curves of the mechanical compression of the orthogonal scaffold without adjacent blocks (n=7).*
